# Self-Perceived Hearing Handicap and Audiometric Severity in Age-Related Hearing Loss: Associations with Age and Sex

**DOI:** 10.3390/audiolres16010024

**Published:** 2026-02-06

**Authors:** Luka Bonetti

**Affiliations:** Department of Hearing Impairments, Faculty of Education and Rehabilitation Sciences, University of Zagreb, Borongajska cesta 83f, 10000 Zagreb, Croatia; luka.bonetti@erf.unizg.hr

**Keywords:** age-related hearing loss, self-perceived hearing handicap, HHIE-S-CRO, pure-tone audiometry, hearing loss severity, multivariate analysis

## Abstract

**Background/Objective**: Self-perceived hearing handicap (SPHH) reflects functional consequences of hearing loss beyond audiometric measures. Clarifying its relationship with audiometric severity and demographic factors is important for understanding age-related hearing loss (ARHL). This study examined associations between SPHH, audiometric measures, age, and sex in individuals with ARHL. **Methods**: A total of 145 adults (50 men, 95 women) aged 60–89 years (mean 71.65 ± 7.19 years) participated. Hearing status was defined using better-ear pure-tone average thresholds at 0.5, 1, 2, and 4 kHz (BE PTA-4), with ≥20 dB HL as the cutoff and World Health Organization (WHO)-defined severity categories. SPHH was assessed using the Croatian Hearing Handicap Inventory for the Elderly–Screening version (HHIE-S-CRO). HHIE-S-CRO total and subscale scores were examined across BE PTA-4 values and hearing loss categories. Associations were analyzed using correlation and linear regression adjusted for age and sex; group differences were tested using the Kruskal–Wallis test, and ordinal logistic regression assessed monotonic trends across ordered severity categories. **Results**: HHIE-S-CRO total and subscale scores increased with worsening BE PTA-4 and across hearing loss categories, with substantial overlap. Strong correlations were observed between HHIE-S-CRO scores and audiometric measures. In linear regression, BE PTA-4 was independently associated with HHIE-S-CRO total, emotional, and social/situational scores, whereas age and sex were not. Kruskal–Wallis tests showed significant differences across hearing loss categories. Ordinal logistic regression anchored to WHO severity categories demonstrated graded associations for HHIE-S-CRO total and emotional scores, while the social/situational subscale showed greater dispersion and overlap despite a statistically significant association. **Conclusions**: SPHH in ARHL shows a strong association with audiometric severity, with particularly robust correspondence for overall and emotional domains, underscoring the complementary role of patient-reported outcome measures alongside audiometric assessment.

## 1. Introduction

Age-related hearing loss (ARHL) is a common condition associated with aging, typically progressing over time as auditory function declines and impairment severity increases [1]. The deficit characteristically begins at high frequencies before extending to mid- and low-frequency regions [2]. ARHL is among the leading causes of disability worldwide and negatively affects quality of life, social interaction, mental health, and cognitive functioning [3]. More than 25% of adults aged ≥60 years meet the World Health Organization (WHO) criterion for disabling hearing loss (better-ear pure-tone average > 35 dB HL) [4]. Global Burden of Disease (GBD)-aligned estimates further demonstrate steep age-related increases in moderate-or-greater hearing loss across populations [4,5]. Despite variation in audiometric definitions and severity thresholds, population-based data consistently identify ARHL as a major public-health concern in aging societies [4,5,6].

Accurate assessment and management of ARHL require consideration of both audiometric status and subjective impact [7]. Because pure-tone audiometry does not fully capture functional and psychosocial consequences, patient-reported outcome measures (PROMs) complement objective testing by capturing communication difficulties, participation restrictions, and psychosocial well-being [8]. Among handicap-focused instruments, the Hearing Handicap Inventory for the Elderly (HHIE) [9] and its 10-item screening version (HHIE-S) [10] are widely used in older-adult screening and clinical research; the HHIE-S was introduced as a brief tool to identify perceived participation restrictions and guide referral for further audiological evaluation [11]. The HHIE-S demonstrates high internal consistency, acceptable test–retest reliability, and meaningful associations with quality-of-life measures [12,13,14]. Although diagnostic accuracy varies with audiometric definitions and population characteristics, the HHIE-S has shown utility for hearing-loss screening and case-finding across clinical and community settings [15,16,17]. Due to its brevity and ease of administration, it is widely used worldwide [18] and has been adapted and validated across multiple languages and cultural contexts [19,20,21,22,23,24,25,26,27].

Comparisons of SPHH with pure-tone audiometry consistently demonstrate imperfect agreement, with subjective difficulty often diverging from audiometric hearing loss [28,29,30,31,32]. This variability is commonly expressed as overlap in SPHH across audiometric severity categories, and SPHH may underestimate early-stage or mild hearing loss [33]. Accordingly, associations between HHIE-S total scores and better-ear pure-tone averages (BE PTA-4) are often only moderate, indicating substantial interindividual differences among individuals with similar thresholds [13]. This divergence is also reflected in false-negative and false-positive classifications when the HHIE-S is used for case finding [34] and in overlap of HHIE-S scores across audiometric categories [27]. Age and sex may further modify hearing-loss reporting and perceived impact, potentially influencing the relationship between SPHH and BE PTA-4 [28,30,32,35]. Concordance and screening sensitivity may vary across age and sex strata in some populations [30,34], and female sex and poorer BE PTA-4 have been linked to higher odds of clinically significant SPHH [36,37].

Accordingly, modeling HHIE-S total and subscale scores jointly with age, sex, and BE PTA-4 may improve interpretability, reduce misclassification in screening contexts, and support more targeted counseling and rehabilitation decisions [27,30,34]. The present study is distinguished by its simultaneous use of WHO-ordered hearing loss severity categories, subscale-specific HHIE-S-CRO outcomes, and demographic adjustment within ordinal regression models to examine graded associations between SPHH and audiometric severity. This approach extends prior HHIE-S research by explicitly evaluating monotonic severity relationships and subdomain-specific behavior in a clinically realistic sample. Against this background, the present study examined the relationship between SPHH, as measured by the Croatian version of the HHIE-S (HHIE-S-CRO) [27], and audiological indicators of hearing loss, with a focus on age-, sex-, and severity-specific patterns of SPHH.

Although associations between HHIE-S scores and pure-tone audiometric measures have been widely reported, comparatively less attention has been paid to how SPHH behaves across ordered WHO hearing loss severity categories when key demographic factors are taken into account. In particular, it remains unclear whether HHIE-S subdomains contribute equally to graded severity differentiation and to what extent interindividual variability and score overlap persist across audiometric categories in clinically realistic samples of previously undiagnosed older adults.

The present study addresses these gaps by: (1) jointly modeling HHIE-S-CRO total and subscale scores with BE PTA-4, age, and sex; (2) testing monotonic, severity-ordered associations anchored to WHO hearing loss categories using ordinal regression; and (3) examining emotional and social/situational subscale behavior across severity categories in an exploratory analysis. Specifically, associations were examined between HHIE-S-CRO total, emotional, and social/situational subscale scores and two indicators of hearing loss: (a) continuous hearing-loss severity quantified by BE PTA-4, and (b) categorical hearing-loss severity defined according to WHO criteria. In addition, the independent and moderating effects of age and sex were evaluated, and interindividual variability in SPHH was characterized across audiometric severity grades.

Building on prior HHIE-S research, the present study explicitly examines HHIE-S total and subscale scores across WHO-defined hearing loss severity categories while adjusting for age and sex, enabling evaluation of graded associations and subscale-specific patterns in a clinically realistic sample of previously undiagnosed older adults.

## 2. Materials and Methods

### 2.1. Recruitment, Inclusion and Exclusion Criteria

The present study used methodological procedures previously described in the HHIE-S-CRO validation study [27], adapted to address the current research objectives.

Data collection took place at the University Hospital Centre Zagreb and the Clinical Hospital Zagreb. Approval to conduct the study was obtained from the relevant institutional ethics committees (Reference Numbers: 34-1/2017 and 01-1094). Hospital administrations authorized a three-month period for participant recruitment and data collection. Throughout the approved study period, outpatients referred by general practitioners from primary care settings for audiological evaluation were approached for participation, with referrals occurring for heterogeneous reasons, including self-reported hearing difficulties, routine age-related screening, and communication concerns raised by family members or healthcare providers. Clinic personnel provided potential participants with written information about the study.

Individuals were eligible for inclusion if they were 60 years of age or older, had been referred for audiological assessment due to health concerns not directly related to hearing (e.g., nasal or throat complaints), and were capable of providing informed consent as well as independently completing the HHIE-S-CRO. The study sample, therefore, represents a clinically realistic population of older adults with previously undiagnosed, unaided hearing status who were referred for ENT evaluation for non-hearing complaints, among whom hearing loss was present but not the primary reason for healthcare contact.

The HHIE-S-CRO comprises 10 self-report items divided equally between domains addressing social/situational listening challenges and emotional responses associated with hearing loss. Each subscale yields a maximum score of 20 points. Item responses are scored on a three-point scale, with higher values assigned to greater perceived difficulty (“Yes” = 4, “Sometimes” = 2, “No” = 0), resulting in a possible total score ranging from 0 to 40. Increasing scores reflect a higher level of SPHH. Both HHIE-S-CRO subscale scores were retained for subsequent analyses examining their relationships with audiometric outcomes and demographic variables.

Participants were excluded from data collection if they were younger than 60 years; had previously undergone audiological testing or received hearing-related counseling; had an established diagnosis of hearing loss; used hearing aids; or demonstrated cognitive, visual, or language-related limitations that could compromise informed consent or the accurate completion of the HHIE-S-CRO. The exclusion criteria were selected to support the objective of assessing the performance of the HHIE-S-CRO against audiological testing, while allowing age and sex to be examined as potential moderating variables. Consistent with methodological approaches used in prior HHIE-S validation research [23,25,26], individuals with prior exposure to hearing loss diagnosis or rehabilitation were not included. This approach reduced the likelihood that prior knowledge, counseling, or intervention would influence self-reported perceptions of social/situational or emotional difficulties, thereby limiting response bias. In addition, excluding previously diagnosed or rehabilitated participants enhanced the ecological validity of the screening process, as the HHIE-S-CRO is routinely administered in clinical settings to individuals with unknown hearing status.

The HHIE-S was originally developed for populations broadly classified as older adults or the elderly, a group historically defined using age threshold of ≥65. Contemporary clinical practice, however, increasingly recognizes individuals aged 60 years and older as an appropriate target for geriatric assessment, particularly in relation to age-associated sensory and functional changes, as this approach reduces the risk of excluding individuals in the early stages of age-related decline while maintaining clinical relevance and sensitivity [38,39]. This operational definition reflects common clinical and epidemiological practice, acknowledging that etiologic confirmation of age-related cochlear change is not feasible in routine clinical samples.

### 2.2. Research Steps

Participant eligibility was verified by clinical staff using a concise screening procedure that incorporated routinely collected demographic information. Data collection began with self-administration of the HHIE-S-CRO questionnaire in a controlled environment designed to minimize noise and visual distraction. Participants subsequently underwent a comprehensive audiological evaluation, including otoscopic examination performed by an audiologist, middle-ear assessment (tympanometry and acoustic reflex testing) conducted by trained nursing staff, and pure-tone audiometry administered by an experienced technician in accordance with ISO 8253-1 standards [40]. All audiological findings were interpreted by a qualified audiologist.

Hearing status was defined using the BE PTA-4 calculated at 0.5, 1, 2, and 4 kHz. A BE PTA-4 value ≥ 20 dB HL was classified as hearing loss, in line with WHO guidelines [4].

### 2.3. Statistical Analysis

Statistical analyses were performed using IBM SPSS Statistics Version 29 (IBM Corporation, Armonk, NY, USA). Data were screened for completeness and distributional characteristics to guide the selection of appropriate statistical methods. Statistical significance was set at *p* < 0.05, with adjustments applied for multiple related comparisons where appropriate.

To characterize interindividual variability and overlap in SPHH, descriptive analyses of HHIE-S-CRO total and subscale score distributions were conducted across BE PTA-4 values and WHO hearing loss categories, focusing on score dispersion and overlap between categories.

Associations between SPHH and audiometric hearing loss were initially examined using Spearman’s rank correlation coefficients between BE PTA-4 and HHIE-S-CRO total and subscale scores. To further quantify these relationships while accounting for demographic factors, linear regression models were fitted with HHIE-S-CRO total and subscale scores specified as dependent variables and BE PTA-4, age, and sex entered as predictors. To examine whether associations differed by demographic characteristics, interaction terms (BE PTA-4 × age and BE PTA-4 × sex) were included in the regression models. Model assumptions were evaluated using standard diagnostic procedures. Although HHIE-S-CRO scores are bounded, linear regression was applied due to the scores’ approximately continuous distribution in this clinical sample and the robustness of linear models to modest departures from normality. Visual inspection of residuals did not indicate marked heteroscedasticity or nonlinearity, and the consistency of findings with nonparametric analyses supported the use of linear regression for these approximately continuous outcomes; alternative modeling approaches for bounded outcomes, such as beta regression, are also conceptually appropriate for HHIE-S-type data.

Differences in HHIE-S-CRO total and subscale scores across WHO-defined hearing loss categories were examined using the Kruskal–Wallis test. To assess graded associations across increasing hearing loss severity while adjusting for age and sex, complementary ordinal logistic regression models were fitted with WHO hearing loss category specified as the ordinal dependent variable and HHIE-S-CRO total or subscale scores entered as predictors, with age and sex included as covariates. In these models, hearing loss category was treated as the dependent variable to quantify the discriminative and monotonic relationship between HHIE-S-CRO scores and audiometric severity, without implying causal direction. This modeling strategy was used solely to evaluate monotonic associations across ordered severity categories and does not imply prediction, diagnosis, or causal direction.

## 3. Results

### 3.1. Participant Characteristics

In total, 145 adults (50 men and 95 women) aged 60–89 years (mean age 71.65 ± 7.19 years) participated. Using the BE PTA-4 ≥ 20 dB HL criterion, 80 participants (29 men and 51 women; mean age 73.19 ± 7.01 years; range 60–89 years) were identified as having hearing loss. In this subgroup, the mean BE PTA-4 was 39.85 ± 11.33 dB HL, with values ranging from 20 to 64 dB HL. The remaining 65 participants (21 men and 44 women; mean age 69.83 ± 6.94 years; range 60–82 years) were categorized as having no hearing loss according to WHO PTA-4 criteria. Participant characteristics by WHO category are shown in Table 1.

### 3.2. HHIE-S-CRO Scores and Audiometric Associations

Age- and sex-stratified descriptive characteristics of participants with hearing loss, including HHIE-S-CRO total and subscale scores, are presented in Table 2. Across age strata, BE PTA-4 values showed an overall age-related increase, with higher audiometric thresholds observed in older age groups. HHIE-S-CRO total scores followed a similar pattern, showing a descriptive increase across age groups.

Within the 60–69-year age group, men reported higher HHIE-S-CRO total scores than women, with higher emotional and social/situational subscale contributions. In contrast, in the 70–79-year group, women reported higher emotional subscale and total HHIE-S-CRO scores than men, while social/situational scores were comparable between sexes. In the 80–89-year group, HHIE-S-CRO total scores were high in both women and men, with slightly higher social/situational scores observed in men.

**Figure 1:**
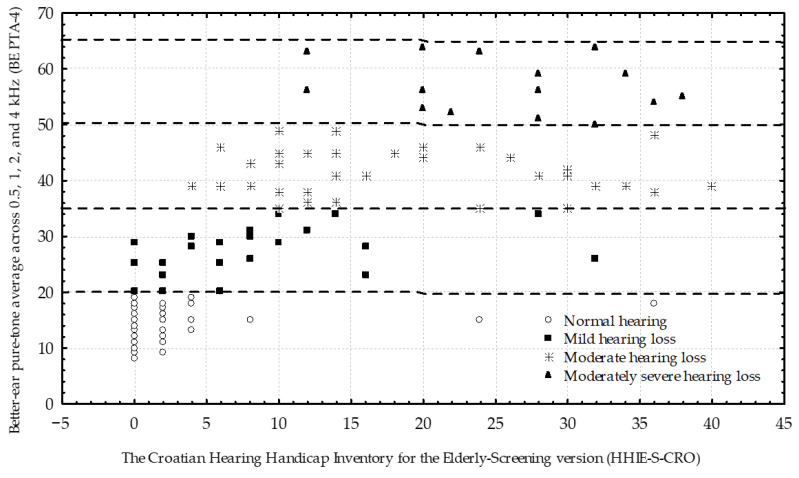
Scatterplot illustrating the association between HHIE-S-CRO total score and BE PTA-4. Horizontal dashed lines indicate WHO hearing loss severity thresholds [4]. Substantial score dispersion and overlap are observed across adjacent WHO hearing loss categories.

Across WHO-defined hearing loss categories, HHIE-S-CRO total and subscale scores showed a clear descriptive increase with worsening audiometric severity (Table 3). HHIE-S-CRO scores were low in participants with no hearing loss according to WHO PTA-4 criteria and increased progressively across mild, moderate, and moderately severe hearing loss categories, with substantial overlap observed between adjacent categories. Figure 1 illustrates the strong positive association between BE PTA-4 and HHIE-S-CRO total score and highlights substantial score overlap across adjacent WHO severity categories.

Stratification by sex revealed broadly comparable HHIE-S-CRO total and subscale scores for women and men within each hearing loss category. In the mild hearing loss category, higher total scores in men were primarily driven by social/situational difficulties, whereas in the moderate hearing loss category, higher total scores in women reflected greater emotional subscale contributions. In participants with moderately severe hearing loss, high HHIE-S-CRO total scores were observed in both sexes, largely driven by social/situational difficulties (Table 3). Overall, emotional and social/situational HHIE-S-CRO subscale scores contributed comparably to total scores descriptively, with social/situational scores tending to exceed emotional scores and with variation in total and subscale scores observed between women and men across hearing loss categories.

Spearman’s rank correlation analyses demonstrated strong positive associations between audiometric hearing loss severity and SPHH. BE PTA-4 was strongly correlated with the HHIE-S-CRO total score (ρ = 0.80, *p* < 0.001), as well as with the emotional (ρ = 0.76, *p* < 0.001) and social/situational (ρ = 0.78, *p* < 0.001) subscale scores.

Table 4 presents the results of the regression analyses with HHIE-S-CRO and its subscales as dependent variables, and BE PTA-4, age, and sex as predictors. In all three models, the multiple correlation coefficients (R), coefficients of determination (R^2^), adjusted coefficients of determination, and the analysis of variance results (F tests) were statistically significant at the selected significance level (*p* < 0.05).

The Durbin–Watson statistic was 1.76 for the regression model with HHIE-S-CRO as the criterion variable, 1.65 for the model with the emotional subscale as the criterion, and 1.98 for the model with the social/situational subscale as the criterion. These values indicate that the regression models were not affected by autocorrelation [41]. The individual contributions of BE PTA-4, age, and sex to the prediction of HHIE-S-CRO scores and their subscales are shown in Table 5, which presents the results of univariate tests of the significance of their relationships with the three self-assessment outcome measures. The significance of the standardized regression coefficients (β) clearly indicates that BE PTA-4 is the variable contributing significantly to the statistical modeling of outcomes.

Partial correlations between BE PTA-4 and the raw scores of HHIE-S-CRO and its subscales are presented in Table 6. These results show that the proportion of unique variance contributed by BE PTA-4 to the prediction of SPHH is substantial: 51.84% for HHIE-S-CRO overall. The audiometric measure was more strongly associated with social/situational consequences of hearing loss (56.25% unique contribution) than with emotional consequences (39.69% unique contribution). The same table also allows evaluation of potential multicollinearity among predictors via tolerance values, with values below 0.10 indicating problematic collinearity [42]. No such issue was observed in the present analyses. No significant BE PTA-4 × age or BE PTA-4 × sex interaction effects were observed for HHIE-S-CRO total or subscale scores (all *p* > 0.45); therefore, results are presented using main-effects models.

Kruskal–Wallis tests revealed that HHIE-S-CRO total scores differed significantly across WHO-defined hearing loss categories (H = 96.39, *p* < 0.001), consistent with progressively greater SPHH with increasing audiometric hearing loss severity. Significant group differences were also observed for both the emotional (H = 85.16, *p* < 0.001) and social/situational (H = 94.34, *p* < 0.001) subscale scores, with higher scores corresponding to more advanced hearing loss categories.

An ordinal logistic regression model with a logit link was used to examine the association between SPHH and WHO-defined hearing loss categories, adjusting for age and sex (Table 7). The final model fit the data significantly better than the intercept-only model (likelihood ratio χ^2^(3) = 107.18, *p* < 0.001). The proportional odds assumption was met (test of parallel lines: χ^2^(6) = 9.37, *p* = 0.154), supporting the use of an ordinal model.

Model fit indices indicated moderate to strong explanatory power (Nagelkerke R^2^ = 0.57, McFadden R^2^ = 0.30). HHIE-S-CRO total score was strongly associated with WHO-defined hearing loss category after adjustment (β = 0.19, SE = 0.02, Wald χ^2^ = 61.54, *p* < 0.001). Each one-point increase in HHIE-S-CRO total score was associated with a 19% increase in the odds of belonging to a more severe hearing loss category (odds ratio = 1.19, 95% CI: 1.14–1.25). This association reflects graded correspondence across ordered severity categories rather than individual-level diagnostic discrimination. Age (β = 0.04, *p* = 0.134) and sex (β = 0.25, *p* = 0.489) were not significantly associated with hearing loss category after adjustment.

A separate ordinal regression model was fitted to examine the association between the emotional HHIE-S-CRO subscale score and WHO-defined hearing loss category. The final model demonstrated significantly improved fit compared with the intercept-only model (χ^2^(3) = 78.12, *p* < 0.001), with the proportional odds assumption satisfied (χ^2^(6) = 9.58, *p* = 0.144). Model fit indices indicated moderate explanatory power (Nagelkerke R^2^ = 0.45, McFadden R^2^ = 0.22). Higher emotional subscale scores were significantly associated with worse hearing status (β = 0.26, SE = 0.04, Wald χ^2^ = 49.16, *p* < 0.001). Each one-point increase in the emotional subscale score corresponded to a 29% increase in the odds of being classified into a more severe hearing loss category (OR = 1.29, 95% CI: 1.20–1.39). Age was also independently associated with hearing loss category in this model (β = 0.06, *p* = 0.017), whereas sex was not (β = 0.46, *p* = 0.184). Despite evidence of data sparsity, deviance-based goodness-of-fit statistics indicated acceptable model fit.

Although the social/situational HHIE-S-CRO subscale showed a strong independent and graded statistical association with WHO-defined hearing loss categories, greater dispersion and overlap of scores across adjacent severity categories were observed, limiting monotonic separation at the individual level. The final model fit was statistically significant (χ^2^(3) = 120.63, *p* < 0.001), with moderate explanatory power (Nagelkerke R^2^ = 0.61, McFadden R^2^ = 0.33). The proportional odds assumption was not violated (χ^2^(4) = 2.67, *p* = 0.615), and both Pearson and deviance goodness-of-fit tests indicated acceptable fit. Age was significantly associated with hearing loss category in this model (β = 0.08, SE = 0.02, Wald χ^2^ = 13.31, *p* < 0.001), whereas sex was not (β = 0.30, *p* = 0.364).

## 4. Discussion

The present study demonstrates a strong and consistent association between SPHH, assessed using the HHIE-S-CRO, and audiometric hearing-loss severity in older adults. Given the cross-sectional design, all findings are interpreted as associations rather than directional or causal relationships. Building on prior HHIE-S studies that often emphasize total-score correlations or dichotomous screening performance, this study explicitly evaluates subscale-specific monotonic associations across WHO severity categories under demographic adjustment, revealing differential behavior of emotional versus social/situational domains.

From a clinical perspective, the present findings reinforce that high levels of self-perceived hearing handicap among individuals with moderate or greater hearing loss are expected and largely reflect advanced functional impairment. In contrast, the more clinically challenging group may be individuals with mild hearing loss, among whom self-perceived handicap shows greater variability. In this group, elevated HHIE-S-CRO scores may signal early psychosocial impact, reduced communicative confidence, or emerging participation restrictions that are not yet fully captured by audiometric thresholds alone, and may therefore warrant particular clinical attention.

Although the present analyses demonstrate graded associations between HHIE-S-CRO scores and audiometric severity, the overlap of scores across adjacent hearing loss categories suggests that fixed cut-off values of HHIE-S-CRO or BE PTA-4 alone may have limited utility for individual-level decision-making. Rather than serving as deterministic thresholds, subjective and objective measures appear most informative when interpreted jointly, particularly for identifying individuals with mild hearing loss who may benefit from early counseling, monitoring, or awareness-raising interventions.

HHIE-S-CRO total and emotional subscale scores showed clear graded associations with WHO-defined hearing loss categories after adjustment for age and sex. In contrast, the social/situational subscale also demonstrated an independent association, but with greater dispersion and overlap across severity categories. This subscale-specific pattern extends prior HHIE-S findings by suggesting differential relationships between perceived emotional burden, situational communication difficulties, and audiometric severity. Notably, the subscale-specific divergence emerged when WHO-ordered severity categories and demographic covariates were modeled simultaneously, which is less commonly made explicit in HHIE-S studies focused primarily on total-score correlations.

Across all analytical approaches, SPHH showed a strong and consistent association with audiometric hearing loss severity. Higher BE PTA-4 values were associated with higher HHIE-S-CRO total scores and with greater emotional and social/situational difficulties, indicating that SPHH increases in parallel with worsening hearing thresholds.

The magnitude of the association observed in the present study (Spearman ρ = 0.80 for the HHIE-S-CRO total score) is notably high and closely matches that reported in prior HHIE-S-CRO validation work using the same BE PTA-4 definition [27]. Comparable studies using the HHIE-S in other linguistic and cultural contexts have reported moderate-to-strong correlations between HHIE-S scores and BE PTA [14,24]. The stronger association observed in the present clinical sample likely reflects a higher prevalence of clinically meaningful hearing loss and a narrower range of unrecognized impairment at presentation. Across studies, HHIE-S scores have been observed to align more closely with pure-tone averages in clinical or help-seeking samples than in population- or community-based screening samples, likely reflecting differences in case mix and awareness of hearing difficulties [15,24,27,34]. The strength of the associations observed in the present study exceeds what is typically reported in population-based or screening samples, which commonly show only moderate correspondence between SPHH and audiometric thresholds, and is likely attributable to the clinic-based, referral sample of older adults with previously undiagnosed hearing loss.

Age was strongly associated with audiometric hearing loss severity in the present sample, with older participants exhibiting higher BE PTA-4 values and a greater prevalence of moderate and moderately severe hearing loss. This finding aligns with the established epidemiology of ARHL, which demonstrates progressive cochlear and neural degeneration with advancing age [2]. Descriptively, HHIE-S-CRO total and subscale scores increased with advancing age, reflecting greater SPHH among older participants. However, when BE PTA-4 was included in multivariable models, age did not independently predict HHIE-S-CRO scores, and no interaction between age and audiometric severity was observed. Together, these findings indicate that age-related differences in SPHH largely reflect underlying audiometric severity rather than an independent effect of age. Similar attenuation of age effects after adjustment for audiometric thresholds has been reported in population-based and clinical studies [29,32]. From a clinical perspective, these findings suggest that SPHH in older adults is most strongly associated with the degree of hearing loss itself rather than with age-related differences in symptom reporting. Nonetheless, age-related factors such as comorbidity burden, reduced cognitive reserve, and diminished compensatory capacity may still influence how hearing loss is experienced and managed in daily life, even if they do not independently alter HHIE-S-CRO scores. A similar pattern was observed for sex: no independent sex differences in SPHH emerged after adjustment for audiometric severity, and no sex-by-severity interactions were detected. This finding suggests that, in later life, the functional impact of hearing loss may be broadly comparable for women and men once degree of hearing loss is taken into account. Although sex-related differences in emotional responses to health conditions have been reported earlier in the adult life course, including a higher prevalence of depressive symptoms among women [43], such differences may attenuate in older age as hearing loss becomes increasingly prevalent and more commonly interpreted as a normative aspect of aging [44]. In addition, selective survival and sex-specific health trajectories in later life may contribute to convergence in self-reported functional impact between women and men [45,46]. Taken together, these findings support the interpretation that SPHH in older adulthood is most strongly associated with audiometric severity itself, with age and sex exerting limited independent influence once hearing loss is taken into account.

At a more granular level, sex-related patterns in SPHH were nuanced. Descriptive analyses revealed variation between women and men in the relative contributions of emotional versus social/situational subscales across age groups and hearing loss categories, suggesting that the experiential profile of hearing-related difficulties may differ by sex. Clinically, this indicates that men and women with comparable audiometric hearing loss report similar overall levels of SPHH, even if the specific ways in which difficulties are experienced or emphasized may differ. This pattern is consistent with previous HHIE-S research showing that sex differences in SPHH are often modest and context-dependent [30,36,37]. Some studies report sex-related differences in SPHH, whereas others report small, null, or reversed sex-related effects depending on sample characteristics and measurement context, making it difficult to generalize about the influence of sex on SPHH [30,36,47,48]. Taken together, these observations support the interpretation that audiometric severity remains the dominant determinant of SPHH within this clinical sample.

The observed sex-related differences in subscale contributions may reflect a combination of sociocultural and psychosocial factors rather than biological differences per se. Social and situational listening demands, as well as norms surrounding communication and help-seeking behavior, may differentially shape how men and women perceive and report the impact of hearing loss. In contrast, emotional responses to hearing difficulties may accumulate over time and be influenced by broader patterns of psychological vulnerability and social roles. These interpretations are necessarily speculative, but are consistent with prior literature suggesting that self-reported health measures are shaped by both sensory impairment and contextual factors.

Analyses based on WHO-defined hearing loss categories demonstrated a clear graded increase in HHIE-S-CRO total and subscale scores with increasing audiometric severity. Participants with moderate and moderately severe hearing loss reported substantially greater SPHH than those with mild or no hearing loss according to WHO PTA-4 criteria, supporting the clinical relevance of WHO severity categories. However, substantial overlap in HHIE-S-CRO scores across adjacent hearing loss categories was evident. This means that individuals with similar audiometric classifications may experience very different levels of perceived impact in everyday life. Such overlap has been consistently documented in HHIE-S studies across diverse populations and languages [15,27,30] and reflects the multifactorial nature of hearing handicap. Beyond pure-tone thresholds, SPHH reflects broader communication-related and psychosocial consequences, including participation restrictions in social life [30,49,50]. Prior work has shown that HHIE-S scores typically explain only a portion of variance in audiometric hearing loss, underscoring that SPHH captures dimensions of hearing disability not reducible to threshold elevation alone [47,49]. From a clinical perspective, the ordinal regression results indicate that HHIE-S-CRO total and emotional subscale scores convey meaningful information about the likelihood of more advanced audiometric hearing loss. Each one-point increase in HHIE-S-CRO total score was associated with a 19% increase in the odds of belonging to a more severe WHO hearing-loss category, underscoring the potential utility of HHIE-S-CRO scores for stratifying the likelihood of more advanced audiometric hearing loss in clinical screening and case-finding contexts. Age showed limited and domain-specific associations across ordinal models. Such variability may reflect domain-specific measurement properties, residual confounding, or contextual influences rather than inconsistent model performance.

Subscale-specific analyses further refined this interpretation. While both emotional and social/situational HHIE-S-CRO scores increased with audiometric severity, the emotional subscale demonstrated a stronger and more consistent monotonic association with WHO-defined hearing loss category after adjustment for age and sex. In contrast, although the social/situational HHIE-S-CRO subscale demonstrated a strong independent and graded association with WHO-defined hearing loss categories, greater dispersion and overlap of scores across adjacent severity categories limited its monotonic separation of individual scores across ordered WHO severity categories. This discrepancy likely reflects the greater context dependence of situational communication difficulties, which may vary substantially across listening environments and therefore scale less monotonically with pure-tone thresholds than emotional responses. As a result, situational difficulties may show strong bivariate associations with hearing loss while exhibiting reduced discriminative performance across ordered severity categories. This suggests that emotional consequences of hearing loss may intensify more predictably as hearing loss worsens, whereas situational difficulties depend more heavily on communication context and environmental demands. Situational listening difficulties are episodic and context-dependent, whereas their emotional consequences may persist and accumulate over time. Emotional self-report items may therefore reflect an integrated impact of repeated listening challenges, while situational difficulties—being intermittent, variable, and subject to adaptation—may be underestimated in self-assessment.

The more prominent role of the emotional subscale observed in the present study is clinically meaningful. Emotional items of the HHIE-S capture internalized reactions to hearing-related communication difficulties, such as embarrassment, frustration, and reduced confidence. These reactions are closely linked to stigma and self-stigma associated with hearing loss and hearing devices [51,52]. Self-stigma related to hearing loss has been shown to contribute to negative self-perception and withdrawal/social segregation [51]. In parallel, hearing-aid stigma remains a major barrier to hearing-aid uptake (non-adoption), despite technological advances [52].

Beyond pure-tone thresholds, SPHH reflects broader functional and psychosocial consequences and is influenced by contextual and individual factors that shape everyday communication and participation [7]. Prior work has noted that situational communication difficulties related to hearing loss are highly context-dependent (e.g., affected by background noise and interactional demands) and may be mitigated by practical communication strategies (e.g., repeating/rephrasing and using written support), which are also central components of structured communication-strategy interventions [53,54]. In contrast, emotional and psychosocial consequences of hearing loss—including reduced confidence, loneliness, and social withdrawal—are widely reported and may not be fully explained exclusively by audiometric thresholds [55]. Both questionnaire-based research and qualitative studies of age-related hearing loss indicate that perceived communication difficulties and psychosocial impact are influenced by contextual and individual factors, underscoring that SPHH also captures non-audible dimensions of hearing disability [7,53].

Taken together, the present findings indicate that the HHIE-S-CRO total score provides a meaningful summary of SPHH that corresponds in a graded manner to audiometric hearing loss severity across age and sex. At the same time, the observed variability and overlap across audiometric categories emphasize that SPHH captures dimensions of hearing disability that extend beyond pure-tone thresholds alone. Clinically, this supports the integration of HHIE-S-CRO assessment with audiometric evaluation to facilitate patient-centered communication, identify individuals experiencing disproportionate impact relative to audiometric severity, and tailor counseling and rehabilitation strategies accordingly. HHIE-S-CRO scores should therefore be interpreted as complementary to, rather than substitutes for, objective audiological measures.

Several limitations should be considered. Because the sample consisted exclusively of previously undiagnosed, unaided older adults recruited from outpatient clinical settings, the present findings may not generalize to individuals with longstanding hearing loss, prior audiological diagnosis, or experience with hearing-aid rehabilitation, nor to population-based screening contexts, where correspondence between self-perceived hearing handicap and audiometric thresholds is typically weaker. Moreover, participants classified as having no hearing loss according to WHO PTA-4 criteria may nevertheless have had high-frequency hearing loss, speech-in-noise deficits, or suprathreshold auditory dysfunction not captured by PTA-4; accordingly, this category should be interpreted as reflecting the absence of WHO-defined hearing loss rather than the absence of auditory dysfunction per se. Second, the cross-sectional design precludes inference regarding temporal or causal relationships between audiometric severity and SPHH. Third, in the present study, ARHL is operationalized pragmatically based on age and audiometric characteristics typical of age-related hearing loss, rather than etiologically confirmed cochlear pathology. Moreover, participants were recruited from outpatient hospital settings, which may limit generalizability to community-dwelling older adults without healthcare contact. Recruitment from hospital outpatient clinics may also have enriched the sample for individuals with unrecognized hearing loss, potentially contributing to the observed strength of associations between audiometric severity and SPHH. However, although the clinic-based sample may limit generalizability to population-based screening contexts, the findings are directly applicable to ENT and audiology outpatient settings where hearing loss is frequently unrecognized at presentation. Third, although exclusion of previously diagnosed or rehabilitated individuals reduced response bias, unmeasured psychosocial or contextual factors may have influenced SPHH. Fourth, reliance on pure-tone audiometry does not capture suprathreshold auditory processing or real-world listening complexity. Finally, as a self-report instrument, the HHIE-S-CRO reflects perceived impact rather than objective performance and should be interpreted accordingly.

## 5. Conclusions

This study addressed its aim by examining SPHH in age-related hearing loss within a multivariate analytical framework, allowing relevant clinical and demographic factors to be considered simultaneously. The findings demonstrate a strong association between SPHH and audiometric hearing loss severity, with particularly robust and consistent relationships observed for overall and emotional domains. By integrating patient-reported outcome measures with audiometric assessment, the study highlights the value of combining subjective and objective information to capture the functional impact of hearing loss. Scientifically, the results underscore the utility of multivariate approaches for investigating complex hearing-related phenomena and offer insight for future research aimed at improving the understanding and management of hearing-related disability. These findings should be interpreted within the context of a cross-sectional, clinic-based sample.

## Figures and Tables

**Table 1:** Participant characteristics stratified by WHO hearing loss categories [4], including sex distribution, age, and BE PTA-4 values.

Hearing Category (BE PTA-4, dB HL)	N (Women/Men)	Mean Age ± SD (Years)	Age Range (Years)	Mean BE PTA-4 ± SD (dB HL)	BE PTA-4 Range (dB HL)
No hearing loss according to WHO PTA-4 criteria (BE PTA-4 ≤ 19 dB HL)	65 (44/21)	69.83 ± 6.94	60–82	14.69 ± 2.89	8–19
Mild hearing loss (BE PTA-4 20 to <35 dB HL)	25 (17/8)	72.32 ± 5.05	60–84	26.96 ± 4.79	20–34
Moderate hearing loss (BE PTA-4 35 to <50 dB HL)	40 (26/14)	72.25 ± 6.98	60–83	41.48 ± 3.94	35–49
Moderately severe hearing loss (BE PTA-4 50 to <65 dB HL)	15 (8/7)	77.13 ± 8.77	60–89	57.00 ± 4.78	50–64

**Table 2:** Mean age, BE PTA-4, and HHIE-S-CRO emotional, social/situational, and total scores in participants with hearing loss, stratified by age group and sex.

Age Group (Years)	N/Sex		Mean Age ± SD (Years)	Mean BE PTA-4 ± SD (dB HL)	Emotional HHIE-S-CRO ± SD	Social/Situational HHIE-S-CRO ± SD	HHIE-S-CRO ± SD
60–69	All	22	64.32 ± 3.01	38.82 ± 11.31	6.82 ± 5.26	8.36 ± 5.78	15.18 ± 10.49
	Women	13	64.23 ± 3.11	36.00 ± 12.75	5.69 ± 4.89	7.54 ± 5.72	13.23 ± 10.08
	Men	9	64.44 ± 3.05	42.89 ± 7.79	8.44 ± 5.64	9.56 ± 5.98	18.00 ± 11.00
70–79	All	44	74.30 ± 2.53	37.93 ± 10.55	6.59 ± 6.55	8.86 ± 5.70	15.45 ± 11.31
	Women	30	74.50 ± 2.52	37.90 ± 11.05	7.67 ± 7.13	8.73 ± 6.11	16.40 ± 12.55
	Men	14	73.86 ± 2.60	38.00 ± 9.79	4.29 ± 4.50	9.14 ± 4.88	13.43 ± 8.05
80–89	All	14	83.64 ± 2.73	47.50 ± 11.33	9.00 ± 5.70	11.86 ± 5.52	20.86 ± 10.92
	Women	8	83.88 ± 2.80	47.00 ± 13.77	9.00 ± 6.76	11.00 ± 6.41	20.00 ± 13.01
	Men	6	83.33 ± 2.88	48.17 ± 8.21	9.00 ± 4.52	13.00 ± 4.34	22.00 ± 8.39

**Table 3:** Mean HHIE-S-CRO emotional, social/situational, and total scores by WHO-defined hearing loss category, stratified by sex.

Hearing Category (BE PTA-4, dB HL)	N/Sex		Emotional HHIE-S-CRO (Mean ± SD)	Social/Situational HHIE-S-CRO (Mean ± SD)	HHIE-S-CRO (Mean ± SD)
No hearing loss according to WHO PTA-4 criteria (BE PTA-4 ≤ 19 dB HL)	All	65	0.62 ± 3.04	1.08 ± 2.55	1.69 ± 5.41
	Women	44	0.59 ± 3.07	1.05 ± 2.64	1.64 ± 5.55
	Men	21	0.67 ± 3.06	1.14 ± 2.41	1.81 ± 5.25
Mild hearing loss (BE PTA-4 20 to <35 dB HL)	All	25	3.12 ± 4.17	5.04 ± 4.80	8.16 ± 8.26
	Women	17	3.29 ± 5.00	4.35 ± 5.21	7.65 ± 9.78
	Men	8	2.75 ± 1.49	6.50 ± 3.66	9.25 ± 3.69
Moderate hearing loss (BE PTA-4 35 to <50 dB HL)	All	40	8.15 ± 6.22	9.75 ± 4.69	17.90 ± 10.21
	Women	26	9.08 ± 6.65	10.08 ± 4.91	19.15 ± 10.88
	Men	14	6.43 ± 5.09	9.14 ± 4.35	15.57 ± 8.71
Moderately severe hearing loss (BE PTA-4 50 to <65 dB HL)	All	15	10.80 ± 4.95	14.93 ± 4.33	25.73 ± 8.07
	Women	8	10.50 ± 5.32	14.00 ± 5.24	24.50 ± 9.96
	Men	7	11.14 ± 4.88	16.00 ± 3.06	27.14 ± 5.64

**Table 4:** Multiple regression models predicting HHIE-S-CRO and its subscale scores from BE PTA-4, age, and sex.

	Multiple Correlation Coefficient (R)	Coefficient of Determination (R^2^)	Adjusted Coefficients of Determination	F Test (df)	Significance (*p*)	Standard Error of Estimate (SE)
HHIE-S-CRO	0.74	0.55	0.54	56.90 (3, 141)	<0.001	7.86
Emotional HHIE-S-CRO	0.64	0.42	0.40	33.43 (3, 141)	<0.001	4.55
Social/situational HHIE-S-CRO	0.78	0.61	0.60	72.95 (3, 141)	<0.001	3.88

**Table 5:** Standardized regression coefficients (β) for predictors of HHIE-S-CRO and its subscales.

	Predictor	Standardized Regression Coefficient (β)	Standard Error of β (SE)	t (141)	Significance (*p*)
HHIE-S-CRO	Sex	−0.01	0.06	−0.13	0.893
	Age	0.05	0.06	0.83	0.407
	BE PTA-4	0.72	0.06	12.18	<0.001
Emotional HHIE-S-CRO	Sex	0.04	0.06	0.64	0.520
	Age	0.01	0.07	0.15	0.881
	BE PTA-4	0.64	0.07	9.51	<0.001
Social/situational HHIE-S-CRO	Sex	−0.05	0.05	−1.03	0.305
	Age	0.08	0.06	1.52	0.134
	BE PTA-4	0.75	0.06	13.50	<0.001

**Table 6:** Partial correlations and unique contributions of BE PTA-4 to the prediction of HHIE-S-CRO and its subscales.

	BE PTA-4				
	Partial Correlation	Unique Contribution to R^2^ (%)	Tolerance	t (141)	Significance (*p*)
HHIE-S-CRO	0.72	51.84	0.91	12.18	<0.001
Emotional HHIE-S-CRO	0.63	39.69	0.91	9.51	<0.001
Social/situational HHIE-S-CRO	0.75	56.25	0.91	13.50	<0.001

**Table 7:** Ordinal logistic regression models of graded association with WHO-defined hearing loss category.

Predictor		Regression Coefficient (β)	Standard Error of β (SE)	Wald χ^2^	Significance (*p*)	Odds Ratio (OR) with 95% Confidence Interval (CI)
Model 1	HHIE-S-CRO	0.19	0.02	61.54	<0.001	1.19 (1.14–1.25)
	Age	0.04	0.03	2.24	0.134	1.04 (0.99–1.09)
	Sex	0.25	0.36	0.48	0.489	1.28 (0.63–2.59)
Model 2	Emotional HHIE-S-CRO	0.26	0.04	49.16	<0.001	1.29 (1.20–1.39)
	Age	0.06	0.02	5.75	0.017	1.06 (1.01–1.11)
	Sex	0.46	0.35	1.76	0.184	1.58 (0.80–3.11)
Model 3	Social/situational HHIE-S-CRO	0.37	0.04	69.76	<0.001	1.45 (1.33–1.58)
	Age	0.02	0.03	0.78	0.376	1.02 (0.97–1.08)
	Sex	−0.02	0.38	0.00	0.962	0.98 (0.47–2.04)

## Data Availability

The data presented in this study are available on request from the corresponding author due to privacy, legal and ethical reasons.

## Abbreviations

The following abbreviations are used in this manuscript:

SPHH	Self-perceived hearing handicap
ARHL	Age-related hearing loss
HHIE-S-CRO	The Croatian adaptation of the Hearing Handicap Inventory for the Elderly–Screening version
BE PTA-4	Better-ear pure-tone average across 0.5, 1, 2, and 4 kHz
WHO	World Health Organization
GBD	Global Burden of Disease
PROMs	Patient-reported outcome measures
HHIE	Hearing Handicap Inventory for the Elderly
HHIE-S	Hearing Handicap Inventory for the Elderly–Screening version
OR	Odds ratio
95% CI	95% confidence interval
dB HL	Decibels hearing level

## References

[1] Britt C.J., Storey E., Woods R.L., Stocks N., Nelson M.R., Murray A.M., Ryan J., Rance G., McNeil J.J., ASPREE Investigators (2024). Age-Related Hearing Loss: A Cross-Sectional Study of Healthy Older Australians. Gerontology.

[2] Wang J., Puel J.-L. (2020). Presbycusis: An Update on Cochlear Mechanisms and Therapies. J. Clin. Med..

[3] Saber H.G., Fatouh F.N., Askoura A., Wassif G.O., Mohamed E.R. (2025). Cognitive Function and Psychological Well-Being in Older Adults with Sensorineural Hearing Loss: An Egyptian Perspective. Clin. Epidemiol. Glob. Health.

[4] World Health Organization (2021). World Report on Hearing.

[5] (2021). GBD 2019 Hearing Loss Collaborators. Hearing Loss Prevalence and Years Lived with Disability, 1990–2019: Findings from the Global Burden of Disease Study 2019. Lancet.

[6] Stevens G., Flaxman S., Brunskill E., Mascarenhas M., Mathers C.D., Finucane M. (2013). Global and Regional Hearing Impairment Prevalence: An Analysis of 42 Studies in 29 Countries. Eur. J. Public Health.

[7] Lazzarotto S., Baumstarck K., Auquier P. Age-Related Hearing Impairment and Impact on Quality of Life: A Review of Available Questionnaires. https://www.jscimedcentral.com/jounal-article-info/Annals%C2%A0of-Otolaryngology-and-Rhinology/Age-Related-Hearing-Impairment--and-Impact-on-Quality-of--Life%3A-A-Review-of-Available--Questionnaires-2992#.

[8] U.S. Food and Drug Administration (2009). Guidance for Industry: Patient-Reported Outcome Measures: Use in Medical Product Development to Support Labeling Claims.

[9] Ventry I.M., Weinstein B.E. (1982). The Hearing Handicap Inventory for the Elderly: A New Tool. Ear Hear..

[10] Lichtenstein M.J., Bess F.H., Logan S.A. (1988). Validation of Screening Tools for Identifying Hearing-Impaired Elderly in Primary Care. JAMA.

[11] National Academies of Sciences, Engineering, and Medicine (2016). Hearing Health Care for Adults: Priorities for Improving Access and Affordability.

[12] Chou R., Dana T., Bougatsos C., Fleming C., Beil T. (2011). Screening Adults Aged 50 Years or Older for Hearing Loss: A Review of the Evidence for the U.S. Preventive Services Task Force. Ann. Intern. Med..

[13] Feltner C., Wallace I.F., Kistler C.E., Coker-Schwimmer M., Jonas D.E. (2021). Screening for Hearing Loss in Older Adults: Updated Evidence Report and Systematic Review for the U.S. Preventive Services Task Force. JAMA.

[14] Tomioka K., Ikeda H., Hanaie K., Morikawa M., Iwamoto J., Okamoto N., Saeki K., Kurumatani N. (2013). The Hearing Handicap Inventory for the Elderly–Screening (HHIE-S) versus a Single Question: Reliability, Validity, and Relations with Quality of Life Measures in the Elderly Community, Japan. Qual. Life Res..

[15] Servidoni A.B., Conterno L.O. (2018). Hearing Loss in the Elderly: Is the Hearing Handicap Inventory for the Elderly–Screening Version Effective in Diagnosis When Compared to the Audiometric Test?. Int. Arch. Otorhinolaryngol..

[16] Calviti K.C.F.K., Pereira L.D. (2009). Sensitivity, Specificity and Predictive Values of Hearing Loss to Different Audiometric Mean Values. Braz. J. Otorhinolaryngol..

[17] Ting H.-C., Huang Y.-Y. (2023). Sensitivity and Specificity of Hearing Tests for Screening Hearing Loss in Older Adults. J. Otol..

[18] Li L.Y.J., Wang S.Y., Wu C.J., Tsai C.Y., Wu T.F., Lin Y.S. (2020). Screening for Hearing Impairment in Older Adults by Smartphone-Based Audiometry, Self-Perception, HHIE Screening Questionnaire, and Free-Field Voice Test. JMIR Mhealth Uhealth.

[19] Lichtenstein M.J., Hazuda H.P. (1998). Cross-Cultural Adaptation of the Hearing Handicap Inventory for the Elderly–Screening Version (HHIE-S) for Use with Spanish-Speaking Mexican Americans. J. Am. Geriatr. Soc..

[20] Jupiter T., Palagonia C.L. (2001). The Hearing Handicap Inventory for the Elderly Screening Version Adapted for Use with Elderly Chinese American Individuals. Am. J. Audiol..

[21] Salonen J., Johansson R., Karjalainen S., Vahlberg T., Isoaho R. (2011). Relationship between Self-Reported Hearing and Measured Hearing Impairment in an Elderly Population in Finland. Int. J. Audiol..

[22] Weinstein B.E., Rasheedy D., Taha H.M., Fatouh F.N. (2015). Cross-Cultural Adaptation of an Arabic Version of the 10-Item Hearing Handicap Inventory. Int. J. Audiol..

[23] Öberg M. (2016). Validation of the Swedish Hearing Handicap Inventory for the Elderly (Screening Version) and Evaluation of Its Effect in Hearing Aid Rehabilitation. Trends Hear..

[24] Wang Y., Mo L., Li Y., Zheng Z., Qi Y. (2016). Analysing Use of the Chinese HHIE-S for Hearing Screening of Elderly in a Northeastern Industrial Area of China. Int. J. Audiol..

[25] Duchêne J., Billiet L., Franco V., Bonnard D. (2022). Validation of the French Version of the HHIE-S Questionnaire in Adults over 60 Years of Age. Eur. Ann. Otorhinolaryngol. Head Neck Dis..

[26] Apa E., Sacchetto L., Palma S., Cocchi C., Gherpelli C., Genovese E., Monzani D., Nocini R. (2023). Italian Validation of the Hearing Handicap Inventory for the Elderly–Screening Version (HHIE-S-It). Acta Otorhinolaryngol. Ital..

[27] Bonetti L., Bonetti A., Krišto T. (2025). Identifying Hearing Loss and Audiological Rehabilitation Candidacy through Self-Perceived Hearing Handicap Using the Croatian Version of the HHIE-S. Audiol. Res..

[28] Choi J.E., Moon I.J., Baek S.-Y., Kim S.W., Cho Y.-S. (2019). Discrepancies between Self-Reported Hearing Difficulty and Hearing Loss Diagnosed by Audiometry. BMJ Open.

[29] Curti S.A., Taylor E.N., Su D., Spankovich C. (2019). Prevalence of and Characteristics Associated with Self-Reported Good Hearing in a Population with Elevated Audiometric Thresholds. JAMA Otolaryngol. Head Neck Surg..

[30] Dillard L.K., Matthews L.J., Dubno J.R. (2024). Agreement between Audiometric Hearing Loss and Self-Reported Hearing Difficulty on the Revised Hearing Handicap Inventory Differs by Demographic Factors. J. Epidemiol. Community Health.

[31] O’Shea B.Q., Milan R.A., Gross A., Powell D.S., Kobayashi L.C., Steptoe A. (2025). Do Self-Reported and Objective Hearing Measures Similarly Relate to General and Domain-Specific Cognition?. BMJ Open.

[32] Tsimpida D., Kontopantelis E., Ashcroft D., Panagioti M. (2020). Comparison of Self-Reported Measures of Hearing with an Objective Audiometric Measure in Adults in the English Longitudinal Study of Ageing. JAMA Netw. Open.

[33] Goman A.M., Reed N.S., Lin F.R., Willink A. (2020). Variations in Prevalence and Number of Older Adults with Self-Reported Hearing Trouble by Audiometric Hearing Loss and Sociodemographic Characteristics. JAMA Otolaryngol. Head Neck Surg..

[34] Zhou X., Fu X., Zhang Y., Zhou J., Cui Y., Liu B. (2025). Optimization of Utilizing the HHIE-S for Hearing Screening in Older People: A Cross-Sectional Study of Associated Factors. Sci. Rep..

[35] Gates G.A., Mills J.H. (2005). Presbycusis. Lancet.

[36] Arnold M.L., Hyer K., Small B.J., Chisolm T., Saunders G.H., McEvoy C.L., Lee D.J., Dhar S., Bainbridge K.E. (2021). Factors Associated with Self-Perceived Hearing Handicap in Adults from Hispanic/Latino Backgrounds. Ear Hear..

[37] Kamil R.J., Genther D.J., Lin F.R. (2015). Factors Associated with the Accuracy of Subjective Assessments of Hearing Impairment. Ear Hear..

[38] McCabe D. (2019). Hearing Screening in Older Adults, Try This: General Assessment 12.

[39] Arora A., Aldridge L., Gordon A. (2024). The State of the Consultant Geriatrician Workforce: An Analysis of the RCP Census.

[40] (2010). Acoustics—Audiometric Test Methods—Part 1: Pure-Tone Air and Bone Conduction Audiometry.

[41] Field A. (2009). Discovering Statistics Using SPSS.

[42] Pallant J. (2007). SPSS Survival Manual: A Step-by-Step Guide to Data Analysis Using SPSS for Windows.

[43] Salk R.H., Hyde J.S., Abramson L.Y. (2017). Gender differences in depression in representative national samples: Meta-analyses of diagnoses and symptoms. Psychol. Bull..

[44] Wettstein M., Reinhard A.-K., Williger B., Wurm S. (2025). Associations of self-reported hearing problems with long-term trajectories of mental and functional health in middle-aged and older adults: The role of self-perceptions of aging. PLoS ONE.

[45] Oksuzyan A., Petersen I., Stovring H., Bingley P., Vaupel J.W., Christensen K. (2009). The male-female health-survival paradox: A survey and register study of the impact of sex-specific selection and information bias. Ann. Epidemiol..

[46] Austad S.N., Fischer K.E. (2016). Sex Differences in Lifespan. Cell Metab..

[47] Gates G.A., Murphy M., Rees T.S., Fraher A. (2003). Screening for Handicapping Hearing Loss in the Elderly. J. Fam. Pract..

[48] Taylor K.S., Jurma W.E. Gender-Specific Audiologic Rehabilitation Programs and Self-Perception of Handicap in the Elderly. https://www.audiologyonline.com.

[49] Humes L.E. (2021). An Approach to Self-Assessed Auditory Wellness in Older Adults. Ear Hear..

[50] Magalhães R., Iório M.C.M. (2011). Quality of Life and Participation Restrictions, a Study in Elderly. Braz. J. Otorhinolaryngol..

[51] da Silva J.C., de Araujo C.M., Lüders D., Santos R.S., de Lacerda A.B.M., José M.R., Guarinello A.C. (2023). The Self-Stigma of Hearing Loss in Adults and Older Adults: A Systematic Review. Ear Hear..

[52] Madara E., Bhowmik A.K. (2024). Toward Alleviating the Stigma of Hearing Aids: A Review. Audiol. Res..

[53] Heffernan E., Withanachchi C.M., Ferguson M.A. (2021). Communication partners’ perspectives of living with someone with hearing loss: A qualitative study. Disabil. Rehabil..

[54] Werther L., Thorén E., Brännström J., Andersson G., Öberg M. (2024). Hearing impaired persons’ experiences with the online Swedish individualized active communication education (I-ACE) program: A feasibility study. Internet Interv..

[55] Shukla A., Harper M., Pedersen E., Goman A., Suen J.J., Price C., Applebaum J., Lin F.R., Reed N.S. (2020). Hearing Loss, Loneliness, and Social Isolation: A Systematic Review. Otolaryngol. Head Neck Surg..

